# Ice in Hospitals

**Published:** 1924-07

**Authors:** Sydney F. Walker


### ICE IN HOSPITALS.

### DISTRIBUTION OF LOW TEMPERATURES.

By SYDNEY F. WALKER.

Ice is produced, and low temperatures generally obtained, by the alternate expansion to the gaseous state and liquefaction of certain substances that lend themselves to the treatment. Nearly all substances may exist in the solid, the liquid, or the gaseous state, the condition they are in at any moment depending upon the pressure to which they are subject and the quantity of heat they have absorbed. Water forms the readiest illustration of this. We know it as water in the liquid state, as ice in the solid state, and as steam or vapour in the gaseous state. Also, we can change its state from one form to another by the addition or subtraction of heat, plus the addition or subtraction of pressure. When passing from one state to another we not only have to change the quantity of heat present, but also the pressure to which the substance is subject. The pressure to which the surface of the substance is subject has an important bearing upon the condition it will assume at any moment. Ice, for instance, when first formed, unless its temperature is below freezing-point, or its surface is subject to pressure, will be sloppy. That portion of the solid matter in the ice that is exposed to the air will tend to melt quite easily, unless its temperature is below freezing-point or it is subject to pressure.

### The Making of Ice.

Ammonia, carbonic acid, and sulphurous acid are the substances usually employed. Ether is sometimes used, and both water and air have been and are used under certain conditions. Ammonia has been employed more frequently than either of the others, largely because of the greater refrigerating effect produced by the evaporation of a certain quantity of the substance than is obtained with the others. The fact that ammonia gives so much higher return for a given expenditure sufficiently accounts for its greater use. The *modus operandi* is the same whatever substance is employed as the refrigerant. A closed circuit, as electrical engineers express it, is formed, and the refrigerant is kept moving through the circuit, changing its condition as it passes from one part of the circuit to another, doing work in one part of the circuit by lowering the temperature of the substances with which it is brought into contact and having work done on it at other parts of the circuit as its own temperature is lowered and the pressure to which it is subject increased. The circuit consists of a condenser, a reservoir of liquid ammonia, or whatever the refrigerant may be, a compressor into which the refrigerant passes after it has done its work, after it has expanded, and expansion coils or the equivalent with connecting pipes. On leaving the expansion coils the refrigerant passes to the inlet valve of the compressor. It is subject in the compressor to whatever pressure may be required, and is then expelled through the delivery valve to the condenser. It passes from the condenser to the reservoir, and thence to the inlet valve of the compressor, and so commences its round again.

### When Ice is Made.

When ice is made for hospital work blocks are formed in tins designed for the purpose. The tins are square in section horizontally, but taper a little, being rather smaller at the bottom than at the top. The object of this is to enable the blocks to be easily removed from the tins. It is usual also when the ice is made in tins in this way to arrange for the water to be stirred for a certain time to prevent the formation of a solid core in the middle of the block. The tins are placed in a tank that has been carefully insulated thermally by lining it with some thermal insulator, such as compressed cork, and a current of cold brine is kept flowing through the tank and around the outside of all the tins. The cold brine has its temperature lowered to the required figure by passing through a vessel in which what are called the evaporating coils are immersed. These evaporating coils form part of the refrigerating circuit; the liquid refrigerant passes into them from the condenser or the reservoir. In the compressor the gas has been subject to the pressure required to enable the cooling water to abstract the latent heat of evaporation from it, and when that is done liquefaction follows. In order that the cooling water shall abstract the latent heat from the gas it should be at a temperature at least five degrees below that of the gas, and it is much better if there is a difference of ten degrees between them.

### The Possibility of Distributing "Cold."

It is quite practicable to distribute "cold," but whether it would be economical is another matter. I understand that it is done in the United States. To distribute "cold" it would be necessary to have a low-temperature generating station, very much on the lines of an electricity generating station, the compressors and evaporators taking the place of the electric generators. It would probably be most convenient to use cold brine for distribution, though direct expansion might be employed in special cases. Transmission of low temperatures is already carried out to a certain extent. I have a case in mind where low temperature has to be transmitted some distance at a hospital, because the engine room where the compressor is situated is away from the main building. Cold brine is employed; its temperature is lowered to the required figure, a little below that to be delivered, and it is pumped to where it is required. In the ships that bring frozen or chilled meat from Australia or South America, for instance, the holds are fitted with brine pipes. The holds are divided into sections, each section being supplied by one or more of these pipes, and the brine is kept in circulation, at whatever velocity may be required, by increasing or decreasing the speed at which the pumps work. By the aid of brine circulation, carefully watched, there should be no difficulty in keeping the temperature of any part of a hospital down to a certain figure.

